# Integrative Proteomic and Transcriptomic Profiling Identifies Candidate Biomarkers for Discriminating Anaphylactic from Cardiac Sudden Death

**DOI:** 10.3390/ijms27052166

**Published:** 2026-02-25

**Authors:** Zhi-hao Fan, Zi-qi Yue, Zi-kang Liu, Zhan-feng Jin, Wei-hua Zhang, He Chen

**Affiliations:** 1 Department of Forensic Medicine, School of Basic Medical Sciences, Harbin Medical University, Harbin 150081, China; 2023020021@hrbmu.edu.cn (Z.-h.F.); yueziqi@hrbmu.edu.cn (Z.-q.Y.); fylzk@outlook.com (Z.-k.L.); jinzf2003@aliyun.com (Z.-f.J.); 2 Department of Pathophysiology, School of Basic Medical Sciences, Harbin Medical University, Harbin 150081, China

**Keywords:** anaphylactic sudden death, proteomics, identification marker, sudden death from coronary heart disease, forensic identification

## Abstract

To address the forensic diagnostic challenge of distinguishing Anaphylactic Sudden Death (ASD) from Sudden Death from Coronary Heart Disease (SD-CHD), this study established *Ldlr^−/−^* mouse models of Atherosclerosis (AS) and ovalbumin-induced Anaphylaxis (AP). LC-MS/MS-based serum proteomic analysis of Atherosclerosis (AS) and Anaphylaxis (AP) mice identified fibronectin 1 (FN1), platelet glycoprotein Ibα chain (GP1BA), and platelet factor 4 (PF4) as candidate biomarkers. These candidates were validated by parallel reaction monitoring (PRM), enzyme-linked immunosorbent assay (ELISA), and immunohistochemistry (IHC) in a combined AS + AP mouse model and in post-mortem human cardiac and bronchiolar epithelial tissue. In mice, serum FN1, GP1BA, and PF4 levels were significantly elevated in the AS group, whereas only FN1 was markedly downregulated in AP mice. In human tissues, FN1, GP1BA, and PF4 were all upregulated in Sudden death from coronary heart disease (SD-CHD) myocardial samples, with FN1 showing the greatest increase. In airway epithelium, FN1 was upregulated in anaphylactic sudden death (ASD) and anaphylactic sudden death (ASD) with Coronary Atherosclerosis (ASD + CAS) groups, while GP1BA was downregulated. These results indicate that FN1 serves as a key differential mouse serum biomarker, while PF4 and GP1BA aid in Sudden death from coronary heart disease (SD-CHD) diagnosis. Collectively, this multimarker, multilevel framework provides a molecular diagnostic strategy for the forensic identification of complex sudden death.

## 1. Introduction

Anaphylactic sudden death (ASD) is a rapidly fatal allergic reaction characterized by sudden respiratory and circulatory collapse, typically occurring within minutes to hours after allergen exposure [1].

In forensic practice, cases of sudden death following intravenous drug administration are frequently encountered. Due to the lack of eyewitnesses and the presence of atherosclerotic lesions in the body, determining the precise cause of death becomes challenging. Differentiating whether death resulted from sudden cardiac death due to coronary heart disease or fatal anaphylactic shock remains a persistent diagnostic difficulty. The diagnosis of anaphylaxis in the postmortem setting involves unresolved challenges in the international literature, due to the complexity of pathogenic factors and pathophysiological processes involved. For forensic autopsies, further problems of differential diagnosis arise, often leaving the forensic pathologist unable to express an opinion with certainty due to the lack of case history, circumstantial, and autoptical–histopathological data [2].

This scenario creates a critical diagnostic dilemma: differentiating between sudden death due to coronary heart disease (SD-CHD) and death from fatal anaphylactic (AP). A pooled analysis of multinational forensic studies (*n* = 215) found that 42.79% of anaphylactic sudden death (ASD) cases were accompanied by coronary atherosclerosis [3,4,5,6,7]. This frequent co-occurrence of two distinct pathologies—each capable of causing rapid death—poses a significant challenge to routine morphological diagnosis and underscores an urgent need for more specific diagnostic tools.

Current diagnostic biomarkers have notable limitations in this differential context. In anaphylactic sudden death (ASD), elevated serum tryptase and IgE, while supportive, are not entirely specific and can be influenced by sampling timing and postmortem changes [8,9,10]. A recent systematic review by Pilia et al. assessed the diagnostic value of postmortem blood tryptase in fatal anaphylaxis. The authors reported a pooled sensitivity of 70% and specificity of 88% across included studies, and identified an optimal cut-off value of 74.2 ng/mL, which yielded a sensitivity of 95.65% and specificity of 88.24% [11]. While these findings support tryptase as a useful adjunct, they also highlight that a negative result does not exclude anaphylaxis, and that optimal diagnostic accuracy depends on appropriate sampling conditions and interpretation within the full forensic context [11]. For Sudden death from coronary heart disease (SD-CHD), conventional myocardial injury biomarkers such as cardiac troponin (cTn) and creatine kinase-MB (CK-MB) serve as an important supplement to morphological examination [12,13]. However, their utility in postmortem settings is severely constrained by confounding factors including (1) the release of these proteins from skeletal muscle or other tissues, leading to potential cross-reactivity and false positives [14], (2) the critical dependence on a precise sampling time window which is often indeterminate after death, (3) postmortem biochemical alterations and redistribution [15]. Most critically, none of these established biomarkers can reliably differentiate between anaphylactic sudden death (ASD) and Sudden death from coronary heart disease (SD-CHD) in the complex, overlapping presentations encountered in forensic casework [16].

In forensic investigations, the postmortem diagnosis of Sudden death from coronary heart disease (SD-CHD) is chiefly determined through a thorough autopsy encompassing both macroscopic and histopathological evaluation of the heart, supplemented by immunohistochemical analysis when indicated [17]. In this setting, serum biomarkers are utilized solely as supplementary references indicators. Despite their diagnostic utility, these biomarkers (cTn, CK-MB) present considerable limitations in both clinical and forensic applications.

High-throughput proteomics, particularly liquid chromatography–tandem mass spectrometry (LC-MS/MS), has emerged as a powerful tool for biomarker discovery, capable of providing comprehensive protein profiles from complex biological samples [18,19]. Its application in forensic science has grown, addressing challenges in areas such as body fluid identification, postmortem interval estimation, and cause-of-death determination [20,21,22,23,24,25]. While these applications have been established primarily in animal models or controlled experimental settings, their translation to routine human forensic casework remains to be validated. Nonetheless, this provides a valuable research framework for our study. To address the specific diagnostic gap between anaphylactic sudden death (ASD) and Sudden death from coronary heart disease (SD-CHD), we designed an integrated cross-species discovery and validation strategy. The primary objective of this study was to identify and validate serum protein biomarkers capable of discriminating between these two causes of sudden death.

Our overall study design was as follows: First, we employed well-established murine models to profile serum proteomic changes under controlled conditions of (i) IgE-mediated anaphylaxis and (ii) progressive atherosclerosis. The use of these animal models allowed for the discovery of protein signatures associated with each pathology in a systematic manner, minimizing the human confounding variables present in retrospective casework. Second, to prioritize findings with translational relevance to human disease, we integrated the murine proteomic data with human peripheral blood transcriptomic datasets from public repositories (GEO: GSE69063 for anaphylaxis; GSE66360 for acute myocardial infarction). Retained are the data where mouse proteomic and human transcriptomic expression trends are consistent, while inconsistent data are filtered out. Finally, the top candidate biomarkers were validated using targeted proteomics (parallel reaction monitoring, PRM), immunoassays (enzyme-linked immunosorbent assay, ELISA), and immunohistochemistry (IHC) on human postmortem samples.

Through this approach, we aimed to test the central hypothesis that an integrated cross-species proteomic and transcriptomic strategy can identify conserved serum protein biomarkers capable of reliably discriminating between anaphylactic sudden death (ASD) and Sudden death from coronary heart disease (SD-CHD) in forensically challenging cases. This work provides a novel molecular framework to aid in the forensic differentiation of complex sudden death, offering a complementary tool to traditional morphological and biochemical analyses.

## 2. Results

### 2.1. Evaluation of Animal Models

**Figure 1:**
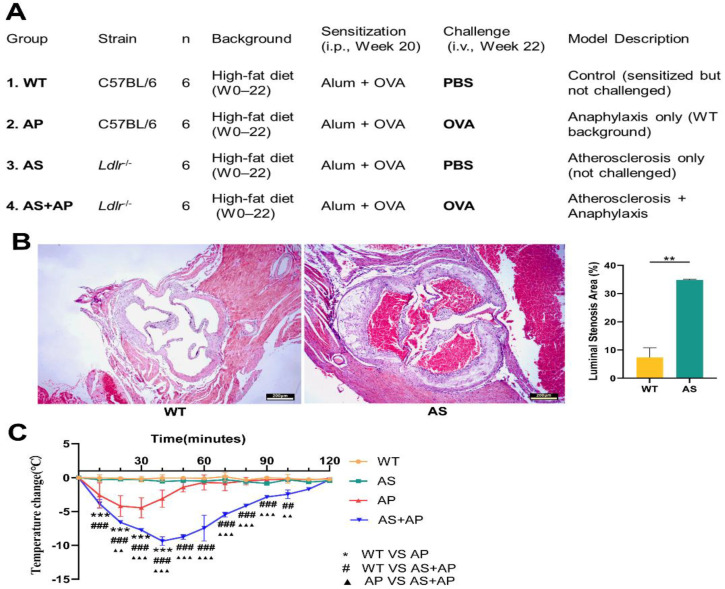
Establishment and evaluation of murine models. (**A**) All mice were fed a high-fat diet for 22 weeks. At weeks 20 and 21, all mice were sensitized with Alum-OVA *i.p.* once a week. At week 22, mice were challenged *i.v.* with PBS (Groups 1 and 3) or OVA (Groups 2 and 4). Group 1 (WT + PBS): control; Group 2 (WT + OVA): anaphylaxis model (AP); Group 3 (*Ldlr^−/−^* + PBS): atherosclerosis model (AS); Group 4 (*Ldlr^−/−^* + OVA): combined model (AS + AP). Serum from Groups 1–3 was used for protein screening; serum/tissues from Group 4 were used for validation. (**B**) H&E-stained aortic root sections (40× magnification) from wild-type (WT) and Ldlr^−/−^ mice, demonstrating atherosclerotic plaque formation and lumen stenosis. (**C**) Rectal temperature changes following tail-vein challenge in WT, AS, AP and AS + AP groups. Data are shown as mean ± SEM (** *p* < 0.01, *** *p* < 0.001; ## *p* < 0.01, ### *p* < 0.001; ▲▲ *p* < 0.01, ▲▲▲ *p* < 0.001).

This study established murine models to identify biomarkers for fatal events associated with anaphylaxis and coronary heart disease. The atherosclerosis model was established in *Ldlr^−/−^* mice fed a high-fat diet (20 weeks, *n* = 12), mimicking human hyperlipidemia and plaque formation—key drivers of sudden cardiac death in coronary artery disease [26]. Control group C57BL/6 (WT) mice were also fed a high-fat diet for 20 weeks (*n* = 12). Starting from week 21, WT mice (*n* = 6) were sensitized by weekly intraperitoneal injections of OVA plus alum adjuvant. Two weeks later, they were challenged with an intravenous OVA injection via the tail vein to induce systemic anaphylaxis (AP group). Similarly, in the *Ldlr^−/−^* group (*n* = 6), sensitization was initiated at week 21 via weekly intraperitoneal injections of OVA (ovalbumin) plus alum adjuvant. Two weeks later, systemic anaphylaxis was induced by an intravenous OVA challenge via the tail vein, thereby establishing the atherosclerosis-plus-anaphylaxis group (AS + AP) (Figure 1A).

To validate the successful establishment of the mouse atherosclerosis model, the mouse arteries were dissected at the aortic root (including the aortic valve region), followed by H&E staining to examine atherosclerotic plaque formation in the arterial wall and assess lumen stenosis. Wildtype (WT) mice showed normal lumen morphology, a smooth intima, and no evidence of atherosclerotic plaque formation. In contrast, the AS group displayed typical atherosclerotic features, including intimal thickening (lumen stenosis rate: 34.83 ± 0.28% vs. WT, *p* < 0.05), foamcell infiltration, and cholesterol crystal deposition (Figure 1B).

Following OVA challenge, compared with WT mice, the AP group exhibited a significant decrease in rectal temperature at 30 min (ΔT = –4.45 ± 1.49 °C, WT vs. AP, *p* < 0.05). The AS + AP group reached the lowest point at 40 min, with a more pronounced temperature drop (ΔT = –9.30 ± 0.57 °C, WT vs. AS + AP, *p* < 0.001). These changes were accompanied by characteristic anaphylactic symptoms such as scratching and tachypnea. These pathological and phenotypic observations collectively confirm the successful establishment of the animal models in all experimental groups (Figure 1C).

### 2.2. Serum Proteomic Profiling Reveals Distinct Molecular Signatures in Atherosclerosis and Allergic Response Models

**Figure 2:**
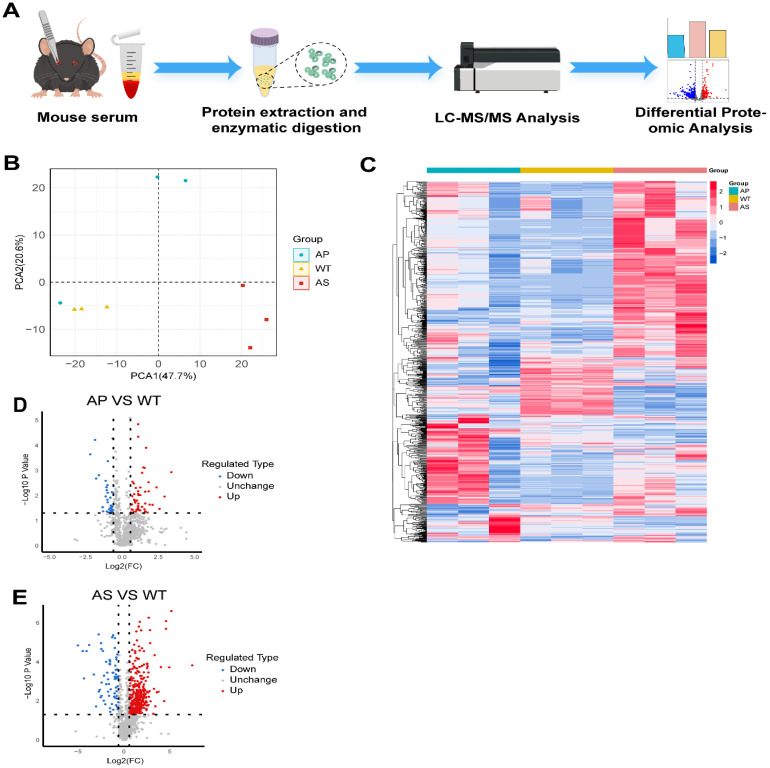
Serum proteomic profiling and functional annotation of differentially expressed proteins in murine models. (**A**) Workflow of serum proteomic analysis. (**B**) Principal component analysis (PCA) of WT, AP, and AS groups, illustrating inter-group variability. (**C**) Hierarchical clustering heatmap of protein expression across groups. (**D**) Volcano plot of differentially expressed proteins in AP vs. WT. (**E**) Volcano plot of differentially expressed proteins in AS vs. WT. DEPs were identified using limma (R v4.3.2) with empirical Bayes moderated *t*-tests and Benjamini–Hochberg (BH) correction (|log_2_fold change (FC)| > 0.58, adjusted *p* < 0.05). WT, wild-type; AP, anaphylaxis; AS, atherosclerosis.

Flowchart of the LC-MS/MS-based proteomic analysis experimental procedure (Figure 2A). LC-MS/MS-based proteomic analysis of individual serum samples from three mice per group (WT, AS, and AP *n* = 3:3:3) identified 9055 peptides, corresponding to 1325 proteins (≥1 unique peptide per protein), with 1317 proteins quantified across all samples for comparative analysis (Table S1). Principal component analysis (PCA) revealed distinct intra-group clustering and significant inter-group separation (Figure 2B), confirming data robustness. Hierarchical clustering analysis revealed distinct protein expression patterns that differentiated all three experimental groups (Figure 2C). Volcano plot analysis identified 74 differentially expressed proteins (47 upregulated and 27 downregulated) in AP versus WT comparisons, and 392 proteins (347 upregulated and 45 downregulated) in AS versus WT comparisons (|log_2_FC| > 0.58, adjusted *p* < 0.05; Figure 2D,E).

Gene Ontology (GO) enrichment analysis revealed that differentially expressed proteins in the AP group were significantly associated with immune response, response to external stimulus, cellular response to stimulus, and antigen binding (Figure S1A). In contrast, differentially expressed proteins in the AS group were enriched for response to stress, regulation of biological quality, and cell communication (Figure S1C). KEGG pathway analysis indicated that AP-associated proteins were involved in TGF-β signaling and graft-versus-host disease pathways (Figure S1B), while AS-associated proteins were enriched in actin cytoskeleton regulation pathways (Figure S1D).

### 2.3. Peripheral Blood Transcriptomic Analysis Identifies 20 Differential Proteins

**Figure 3:**
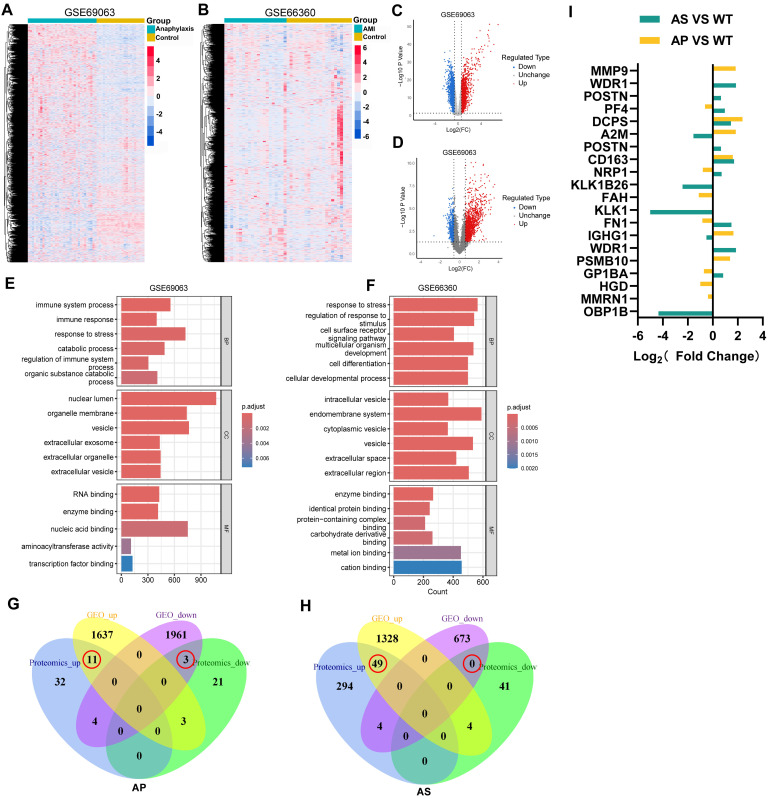
Human peripheral blood transcriptomic analysis. (**A**,**B**) Hierarchical clustering heatmaps of the GSE69063 (**A**) and GSE66360 (**B**) datasets, showing distinct expression profiles. (**C**,**D**) Volcano plots of the GSE69063 (**C**) and GSE66360 (**D**) datasets, highlighting significantly differentially expressed genes. (**E**,**F**) Gene Ontology (GO) enrichment analysis of the GSE69063 (**E**) and GSE66360 (**F**) datasets, showing enriched terms in biological processes, cellular components, and molecular functions. (**G**,**H**) Venn diagrams of the anaphylaxis (AP) (**G**) and atherosclerosis (AS) (**H**) groups, illustrating overlaps between proteomic and transcriptomic data. (**I**) Bar plots of proteomic expression differences: AS vs. wild-type (WT) (green) and AP vs. WT (yellow). Bars indicate significantly differential proteins (*p* < 0.05).

To validate the generalizability of the findings, two independent human peripheral blood transcriptomic datasets were analyzed, representing anaphylaxis (GSE69063) and acute myocardial infarction (AMI, GSE66360). Unsupervised hierarchical clustering revealed distinct disease-specific transcriptional signatures that clearly discriminated each condition from healthy controls (Figure 3A,B). Volcano plot analysis identified 3619 differentially expressed genes (DEGs; 1651 up-regulated, 1968 down-regulated) in the anaphylaxis dataset GSE69063 (Figure 3C) and 2058 DEGs (1381 up-regulated, 677 down-regulated) in the acute myocardial infarction (AMI) dataset GSE66360 (|log_2_FC| > 1, adj *p* < 0.05) (Figure 3D). GO enrichment analysis of DEGs from GSE69063 highlighted immune-related pathways, including immune system process (GO:0002376), immune response (GO:0006955), and regulation of immune system process (GO:0002682), along with extracellular exosome (GO:0070062) (Figure 3E). In contrast, DEGs from GSE66360 showed enrichment in cell surface receptor signaling pathway (GO:0007166), endomembrane system (GO:0012505), and enzyme binding (GO:0019899) (Figure 3F). Integrated Venn analysis of transcriptomic and proteomic data identified 14 proteins (11 up-regulated, 3 down-regulated) in the anaphylaxis (AP) group and 49 proteins (all up-regulated) in the atherosclerosis (AS) group, with consistent expression patterns across both omics layers (Figure 3G,H).

Through an integrated analysis combining Venn selection, GO/KEGG enrichment, and expression thresholds (|log_2_FC| > 1, adj *p* < 0.05), we ultimately identified 20 high-confidence differential proteins. Of these, 15 were significantly dysregulated in the AS model (10 up, 5 down) and 13 in the AP model (6 up, 7 down), with an overlap of 8 proteins between the two conditions. Notably, four proteins (GP1BA, FN1, NRP1, PF4) displayed a reciprocal regulatory pattern—upregulated in AS but downregulated in AP—suggesting model-specific responses. In contrast, IGHG1 and A2M were downregulated in AS and upregulated in AP, while CD163 and DCPS were significantly upregulated in both groups (Figure 3I).

### 2.4. Two-Step Validation in Mouse Serum Conclusively Identifies GP1BA, FN1, and PF4 as Biomarkers

**Figure 4:**
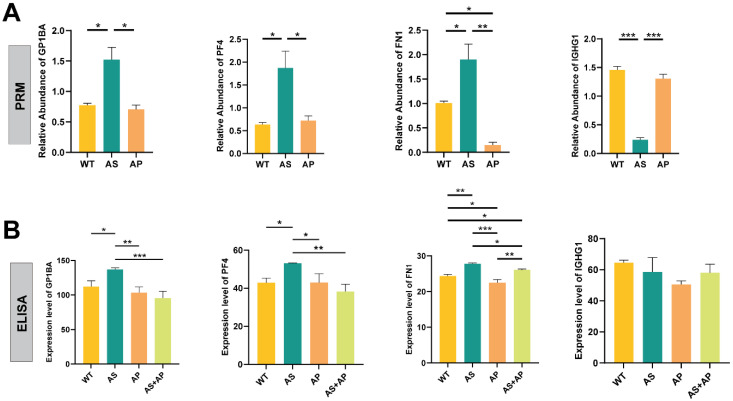
Validation of differentially expressed candidate proteins. (**A**) Relative abundances of target proteins measured by parallel reaction monitoring (PRM) in serum samples from wild-type (WT), atherosclerosis (AS), and anaphylaxis (AP) mice. (**B**) Serum levels of FN1, GP1BA, PF4, and IGHG1 in WT, AS, AP, and AS + AP groups quantified by ELISA. Data are presented as mean ± SEM. Significant differences between groups are indicated. * *p* < 0.05, ** *p* < 0.01, *** *p* < 0.001.

A two-step validation strategy was employed to verify the candidate proteins. First, parallel reaction monitoring (PRM) was performed on mouse serum to assess the 20 selected proteins. Significant dysregulation of several targets was confirmed, including the upregulation of GP1BA, FN1, and PF4 in the AS group. Additionally, FN1 was found to be downregulated in the AP group, while IGHG1 was specifically downregulated in the AS group, which was consistent with the initial proteomic predictions (Figure 4A).

Based on these results, four proteins (GP1BA, FN1, PF4, and IGHG1) were selected for further orthogonal validation. Enzyme-linked immunosorbent assay (ELISA) was conducted on a separate serum cohort. The upregulation of GP1BA, FN1, and PF4 in the AS group, along with the downregulation of FN1 in the AP group, was corroborated by ELISA. Furthermore, an intermediate serum level of FN1 was observed in the AS + AP group, which was statistically distinct from both the AS and AP groups. In contrast, no significant differential expression of IGHG1 was detected across groups by this method (Figure 4B).

### 2.5. Histopathological Validation in Human Tissues

Immunohistochemical validation was performed on myocardial tissue from a post-mortem human cohort to assess the expression of three candidate proteins (FN1, GP1BA, PF4). The cohort consisted of 20 cases equally divided into four groups: healthy individuals who died of accidental injury (Control), sudden coronary heart disease death (SD-CHD), anaphylactic shock death (ASD), and combined anaphylaxis with coronary atherosclerosis (ASD + CAS). Demographic characteristics, including age and sex, were comparable across all groups (*p* > 0.05; Table 1 and Table 2).

**Figure 5:**
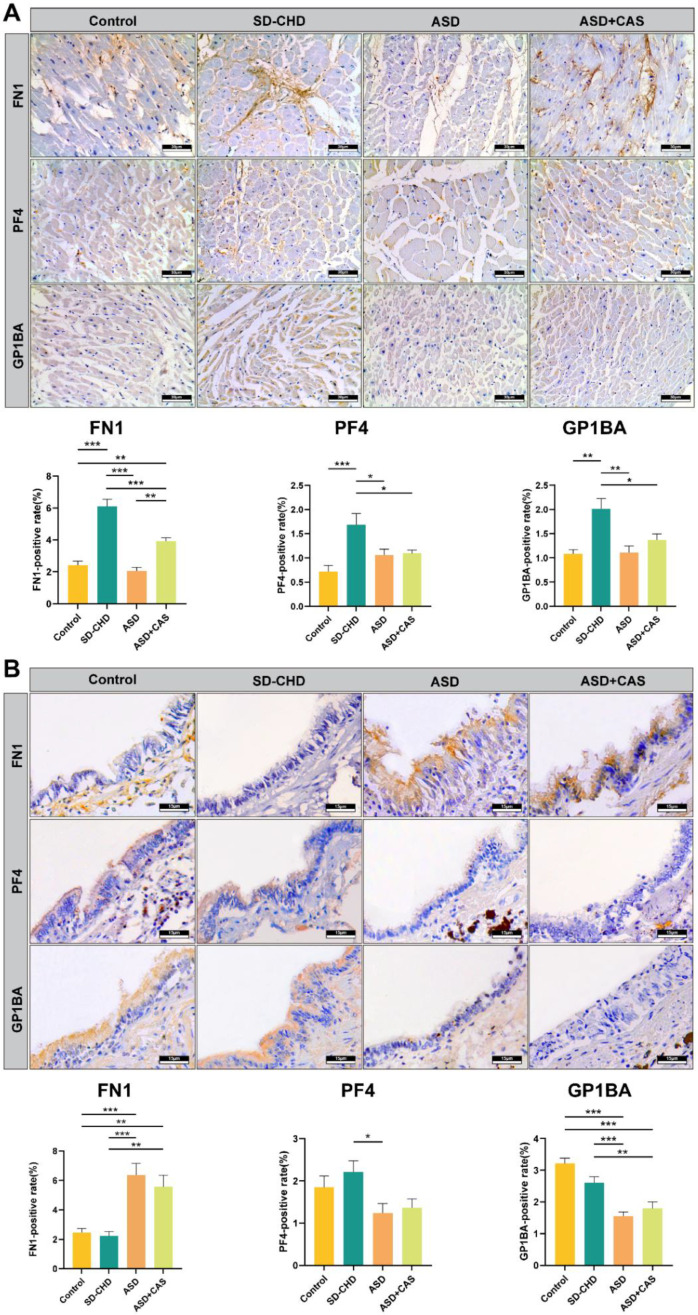
Immunohistochemical validation of FN1, GP1BA, and PF4 expression in human post-mortem tissues. (**A**) Representative IHC images and quantitative analysis of myocardial tissue from Control, SD-CHD, ASD, and ASD + CAS groups (*n* = 5 per group). In cardiac tissue, FN1 was primarily localized to the extracellular matrix, with interstitial deposition significantly increased in the SD-CHD group (*** *p* < 0.001) and moderately elevated in the ASD + CAS group (** *p* < 0.01) compared to Controls. Both PF4 and GP1BA were detected in the cytoplasm of cardiomyocytes and were significantly upregulated in the SD-CHD group (Bars: 30 µm). (**B**) Representative IHC images and quantification of bronchiolar epithelial tissue from the same cohort. In airway epithelium, FN1 expression was significantly upregulated in the ASD and ASD + CAS groups (*** *p* < 0.001), while GP1BA was downregulated in these groups (*** *p* < 0.001). No significant change in PF4 expression was observed in any group. Data are presented as mean ± SD; statistical significance is indicated as * *p* < 0.05, ** *p* < 0.01, *** *p* < 0.001, bars: 15 µm.

IHC analysis revealed distinct localization patterns: FN1 was primarily deposited in the extracellular matrix, while PF4 and GP1BA signals were localized to the cytoplasm of cardiomyocytes. Compared to the healthy Control group, interstitial FN1 deposition was significantly elevated in the SD-CHD group (*p* < 0.001) and moderately increased in the ASD + CAS group (*p* < 0.01), but unchanged in the ASD group. Both PF4 and GP1BA were significantly upregulated in the SD-CHD group (*p* < 0.001) compared to Controls (Bar: 30 μm, Figure 5A).

Immunohistochemical analysis of human bronchiolar epithelial tissue was performed to validate the expression of the three candidate proteins (FN1, GP1BA, PF4). Distinct tissue-specific expression profiles were observed. Compared to the Control group, FN1 expression was significantly upregulated in the ASD and ASD + CAS groups (*** *p* < 0.001), while no significant change was detected in the SD-CHD group. In contrast, GP1BA expression was found to be consistently downregulated in the ASD and ASD + CAS groups (*** *p* < 0.001), with its level remaining unchanged in the SD-CHD group. No significant differential expression of PF4 was observed in any patient group within this tissue. These results collectively highlight a marked divergence in the expression regulation of FN1, GP1BA, and PF4 between cardiac and airway tissues (Bar: 15 μm, * *p* < 0.05, ** *p* < 0.01, *** *p* < 0.001; Figure 5B).

Given the limitations of post-mortem serum markers, we performed immunohistochemistry (IHC) to validate the tissue expression of three candidate proteins (FN1, GP1BA, PF4) in mouse myocardial and airway epithelium. Positive expression was indicated by brown granules in respective cellular compartments. In myocardial tissue, all three proteins were significantly upregulated in the AS + AP and AS groups compared to WT, but not in the AP group. In airway epithelium, FN1 was upregulated in the AS + AP and AP groups, while GP1BA and PF4 were downregulated (Bar: 30 μm, * *p* < 0.05, ** *p* < 0.01, *** *p* < 0.001; Figure S2).

## 3. Discussion

This study integrates proteomics, transcriptomics, and multi-platform validation to systematically delineate the differential expression profiles of FN1, PF4, and GP1BA in cases of anaphylactic sudden death and sudden death from coronary heart disease. Its primary significance lies in addressing the critical forensic challenge of diagnosing acute anaphylaxis in individuals with severe pre-existing coronary atherosclerosis, where rapid demise often leaves insufficient morphological evidence. Our key contribution is the identification of a serum biomarker panel with diagnostic potential and, crucially, the translation of these circulating markers into stable, traceable histopathological signatures via immunohistochemistry. This work establishes a coherent evidence chain—from molecular discovery to practical application—for distinguishing anaphylactic sudden death from sudden cardiac death in forensically complex cases.

We use the terms “anaphylactic sudden death (ASD)” and “sudden death from coronary heart disease (SD-CHD)” operationally to define our two distinct study cohorts. Our goal is to identify discriminative molecular markers to aid in postmortem differentiation between these common scenarios, not to define their ultimate pathological causes.

It is known that when allergen exposure is confirmed, forensic pathologists typically evaluate peripheral blood tryptase to determine if anaphylaxis has occurred. However, while postmortem tryptase is a standard confirmatory test, its utility is limited by strict sampling constraints and degradation over time. Most crucially, it cannot differentiate anaphylaxis from other sudden death causes like acute coronary events. Our study therefore seeks complementary, stable biomarkers for this specific differential diagnosis. Fibronectin (FN1), a glycoprotein synthesized primarily by vascular endothelial cells and hepatocytes, is integral to processes including cell migration, wound healing, and fibrogenesis [27,28,29,30]. Its diagnostic value in sudden death lies in its distinct organ-specific expression patterns. In sudden death from coronary heart disease (SD-CHD), FN1 deposition increases markedly within the myocardial extracellular matrix [31], reflecting a fibrotic reparative response to ischemic injury mediated through pathways such as TGF-β1–Smad2/3 and integrin α5β1–FAK–PI3K–Akt [32,33,34,35]. Conversely, in anaphylactic sudden death (ASD), FN1 is specifically upregulated in the pulmonary airway epithelium, where it contributes to acute airway remodeling under the induction of Th2 cytokines (IL-4/IL-13) via the STAT6 pathway [36,37,38]. Notably, the observed downregulation of serum FN1 in allergic models—which contrasts with its tissue upregulation—underscores the necessity of integrating multidimensional (serum and tissue-based) evidence in comprehensive forensic diagnostics.

PF4 and GP1BA, as platelet-related proteins, play distinct yet complementary roles in thrombosis and disease-specific pathology [39,40,41,42]. PF4, stored in platelet α-granules, not only serves as a marker of acute platelet activation but also promotes atherosclerosis by facilitating oxidized LDL uptake by macrophages [43]. Its elevated levels in both atherosclerotic models and acute myocardial infarction patients further underline its involvement in plaque instability and thrombotic events [44]. GP1BA exhibits a bidirectional, disease-specific expression pattern: it is upregulated in cardiomyocytes in Sudden death from coronary heart disease (SD-CHD), supporting its role as a marker of myocardial injury and coronary thrombosis [45], while being significantly downregulated in pulmonary and myocardial tissues in anaphylactic sudden death (ASD), suggesting a distinct, allergy-associated suppression.

This study employed a cross-species, multi-omics integrative strategy to enhance biomarker discovery and translational relevance. Proteomic screening in mouse models was combined with human transcriptomic databases, followed by functional enrichment and network analysis to identify mechanistically relevant candidates. A “mouse-discovery–human-validation” dual-track approach was then implemented, confirming FN1, GP1BA, and PF4 as discriminative biomarkers through parallel validation in human autopsy blood, cardiac, and airway tissues. This framework establishes a transferable biomarker system for sudden death differentiation, offering a molecular-pathological strategy for forensic identification of complex sudden-death cases. The present study systematically delineated the differential expression profiles of FN1, GP1BA, and PF4 through an integrated multi-layer strategy. From an initial discovery of 286 differentially expressed proteins in mouse serum proteomics, cross-referencing with human transcriptomic datasets (GSE69063 and GSE66360) retained 20 candidates with concordant dysregulation. Targeted validation by PRM and ELISA confirmed FN1, GP1BA, and PF4 as robust candidates, and final validation in human autopsy tissues demonstrated that these three proteins exhibited consistent, disease-specific expression patterns across animal models, human transcriptomes, and human protein levels. This multi-layer concordance provides a solid molecular foundation for their potential application in forensic differential diagnosis.

Notably, FN1 exhibited striking tissue-specific expression patterns: it was significantly upregulated in myocardial tissue of the SD-CHD group, but elevated in pulmonary tissue of the ASD group. This dichotomy aligns with the distinct pathophysiology of each condition—myocardial fibrosis following ischemic injury requires extracellular matrix remodeling mediated by FN1 [31], while anaphylaxis-induced airway remodeling involves FN1 upregulation in bronchial epithelium via Th2 cytokine signaling (IL-4/IL-13) [36,37,38]. These findings suggest that the diagnostic value of FN1 lies not only in its expression level but also in its tissue distribution: myocardial FN1 upregulation points toward SD-CHD, whereas pulmonary FN1 upregulation indicates ASD. GP1BA exhibited a more complex bidirectional regulation: it was upregulated in myocardial tissue of the SD-CHD group but downregulated in both myocardial and pulmonary tissues of the ASD group. As a platelet membrane glycoprotein (GpIbα), GP1BA tissue levels may reflect disease-specific platelet–tissue interactions—platelet activation and microthrombus formation in SD-CHD lead to myocardial GP1BA deposition, while in anaphylaxis, platelets may be recruited to the pulmonary vasculature and degranulate, resulting in decreased myocardial GP1BA levels. This previously unreported phenomenon in anaphylaxis warrants further investigation into platelet dynamics during fatal allergic reactions.

The findings of this study must be contextualized within the practical constraints and needs of forensic pathology. As rightly emphasized, the worldwide gold standard for diagnosing SD-CHD remains the integration of cardiac macroscopic examination, histopathology, and immunohistochemistry [17], while anaphylaxis diagnosis combines circumstantial evidence with biochemical support (e.g., tryptase) [46]. Our goal is not to supplant these essential methodologies but to propose a complementary biochemical tool for the specific and not-uncommon scenario. While tryptase remains a cornerstone of biochemical anaphylaxis diagnosis, the biomarkers identified in this study (FN1, GP1BA, and PF4) are proposed as complementary tools to aid in differential diagnosis, especially in cases where traditional markers are inconclusive or when additional molecular evidence is required.

FN1, GP1BA, and PF4 reflect distinct pathophysiological dimensions of ASD and SD-CHD: FN1 indicates tissue remodeling (myocardial fibrosis vs. airway remodeling), PF4 reflects platelet activation and atherosclerotic plaque instability, and GP1BA suggests disease-specific alterations in platelet–tissue interactions. The combined assessment of these three markers may therefore enhance diagnostic accuracy by capturing multiple pathological facets. This is particularly valuable in hyperacute deaths (<6 h) where morphological evidence is lacking. For example, in a case with antibiotic exposure history but no definitive morphological findings, myocardial FN1 and PF4 upregulation without pulmonary FN1 elevation would support SD-CHD; conversely, pulmonary FN1 upregulation with myocardial GP1BA downregulation would support ASD. Future studies with larger cohorts are warranted to establish a quantitative multimarker discriminant model and validate its diagnostic performance.

This study, however, has several limitations. First, the sample size should be expanded to validate the diagnostic model in larger cohorts. Second, the findings remain largely observational; the specific mechanistic role of FN1 in allergic airway remodeling and the causal basis for GP1BA down-regulation in anaphylactic sudden death (ASD) pulmonary tissue require functional validation in cellular or animal models. Third, the cellular source of GP1BA in tissues—for instance, whether derived from platelet deposition or epithelial internalization—needs to be clarified using approaches such as immunofluorescence co-localization. It is important to note that many of the proposed biomarker signatures, including those from key studies cited here, have been identified and validated primarily in controlled animal models of disease. While these models provide invaluable mechanistic insights, the translation and validation of these findings into the complex, heterogeneous setting of human forensic casework—with its inherent variables such as postmortem interval, agonal processes, and co-morbidities—remains a significant and necessary challenge. The ‘overcoming’ of this translational gap represents a critical next step for the field.

Our study employs controlled models of sub-lethal anaphylaxis and atherosclerosis. This design is intentional and directly addresses the common forensic “gray zone” where death occurs without definitive lethal-grade pathology, such as in cases of severe coronary stenosis without acute occlusion or allergen exposure without classic anaphylactic signs. We hypothesize that even at sub-lethal stages, these distinct conditions trigger unique and detectable molecular pathways. The proteomic signatures identified here (e.g., FN1, PF4, GP1BA), which differentiate an acute allergic response from acute coronary stress in our models, are therefore not direct “causes of death.” Instead, they represent discriminative molecular mechanisms. If validated in human forensic cases, such markers could provide crucial objective evidence to clarify which pathological mechanism was active at the time of death, thereby aiding cause-of-death determination when traditional autopsy findings are inconclusive.

Moreover, a potential future direction for the clinical application of our identified markers (FN1, PF4, GP1BA) would be to evaluate their performance within a composite biomarker panel alongside established biomarkers such as cardiac troponin and CK-MB. Given that FN1 is implicated in fibrosis and PF4 in platelet activation, they may provide complementary pathophysiological information distinct from the myocardial necrosis indicated by troponin. Strategically combining these markers could potentially enhance diagnostic sensitivity in early disease phases or improve risk stratification for adverse cardiac events, beyond what is achievable with current standard biomarkers alone. Future studies are warranted to validate the additive or synergistic value of such a multimarker approach.

In summary, our study established a translational cross-species filtering strategy by combining mouse model proteomics and human database analysis to identify candidate markers with potential applicability in human tissues. Although the current candidates require further large-sample validation in human tissues, the methodological framework provides a feasible and efficient approach for future marker discovery in similar translational research scenarios, especially when direct human samples are limited at the discovery stage.

Future studies could explore the following directions: (1) developing and prospectively validating a multi-parameter diagnostic scoring system that integrates the biomarkers described here; (2) applying spatial multi-omics technologies to precisely characterize the in -situ microenvironment associated with abnormal expression of these markers; and (3) investigating whether therapeutic modulation of these targets can alter disease progression, thereby establishing their mechanistic importance.

## 4. Materials and Methods

### 4.1. Experimental Animals

All animal procedures complied with the ARRIVE guidelines and were approved by the Institutional Animal Care and Use Committee (IACUC) of Harbin Medical University (Approval No. HMUIRB2022007), following the International Guiding Principles for Biomedical Research Involving Animals. Mice were maintained in a specific pathogen-free (SPF) facility under controlled conditions at constant temperature (24 °C) and humidity (40%), with a fixed light/dark cycle (8:00 AM–8:00 PM).

### 4.2. Animal Modeling

Six-week-old male C57BL/6 (wild-type, WT) and low-density lipoprotein receptor gene knockout (*Ldlr^−/−^*) mice (twelve each), obtained from Changzhou Cavens Laboratory Animal Co., Ltd. (Changzhou, Jiangsu, China), were fed a high-fat diet (HFD; 15% fat, 1.25% cholesterol) for 20 weeks to establish atherosclerosis (AS) models [45]. Starting in week 21, an active sensitization protocol was implemented: mice received intraperitoneal injections of a sensitization cocktail (50 μg ovalbumin plus 40 mg aluminum hydroxide adjuvant in 0.2 mL PBS) on days 0 and 7. On day 14, mice were administered a tail-vein injection of either 100 μg of ovalbumin (OVA) (Yeasen Biotechnology (Shanghai) Co., Ltd., Shanghai, China) dissolved in 0.2 mL of phosphate-buffered saline (PBS) to induce anaphylaxis, or an equivalent volume of PBS as a control [47]. The study included four experimental groups: (i) wild-type (WT) mice receiving PBS (WT control); (ii) WT mice receiving OVA (anaphylaxis, AP group); (iii) AS mice receiving PBS (AS group); and (iv) AS mice receiving OVA (AS + AP group). Each group consisted of six mice (*n* = 6). Mice were monitored for behavioral changes and rectal temperature at 5-min intervals for 120 min post-challenge. Terminal blood samples were collected via orbital puncture under anesthesia (mice were anesthetized by intraperitoneal injection of 2% pentobarbital sodium at 40 mg/kg body weight), followed by euthanasia through cervical dislocation. After clotting at room temperature for 30 min, blood was centrifuged (3000× *g*, 15 min, 4 °C) to obtain serum, which was stored at −80 °C. Cardiac and lung (bronchiolar) tissues were fixed in 4% paraformaldehyde. This study was performed in line with the principles of the Declaration of Helsinki. Approval was granted by the Ethics Committee of Harbin Medical University (No. HMUIRB2022007, 13 June 2022).

### 4.3. Proteomic Analysis

#### 4.3.1. Sample Preparation

Serum samples pooled from wild-type (WT), AP, and AS model mice (*n* = 3 per group) were thawed on ice and centrifuged (12,000× *g*, 10 min, 4 °C) to remove cellular debris. The supernatant was transferred to new tubes, and the total protein concentration was quantified using a BCA assay kit (Shanghai Beyotime Biotechnology Co., Ltd., Shanghai, China). Equal amounts of protein from each sample were subjected to enzymatic digestion. The volume was normalized with lysis buffer, followed by reduction with 5 mM dithiothreitol (DTT) (Beijing Biolab Technology Co., Ltd., Beijing, China) at 56 °C for 30 min. Alkylation was performed using 11 mM iodoacetamide (IAA) (Beijing Solarbio Science & Technology Co., Ltd., Beijing, China) in the dark at room temperature for 15 min. The alkylated samples were transferred to ultrafiltration units and centrifuged (12,000× *g*, 20 min, room temperature). Three washes with 8 M urea were performed, followed by three buffer-exchange washes. Digestion was carried out overnight with trypsin at an enzyme-to-protein ratio of 1:50 (*w*/*w*). Peptides were recovered by centrifugation (12,000× *g*, 10 min, room temperature), and a second elution was performed with ddH_2_O. The resulting peptide solutions were combined for subsequent analysis.

#### 4.3.2. LC-MS/MS Analysis

Peptides were reconstituted in mobile phase A (0.1% formic acid, 2% acetonitrile in water) and separated using a Vanquish Neo UHPLC system (Thermo Fisher Scientific Inc., Waltham, MA, USA). Mobile phase B consisted of 0.1% formic acid in 90% acetonitrile/water. The gradient was programmed as follows: 0–16 min, 7–20% B; 16–24 min, 20–32% B; 24–27 min, 32–80% B; 27–30 min, 80% B, at a constant flow rate of 500 nL/min. The separated peptides were ionized via nano-ESI (2.3 kV) and analyzed using FAIMS (compensation voltages: −45 V and −70 V), followed by detection with an Orbitrap Exploris 480 mass spectrometer (Thermo Fisher Scientific Inc., Waltham, MA, USA). Full MS scans were acquired at a resolution of 30,000 over an *m*/*z* range of 390–810, and MS/MS scans were performed with a fixed start *m*/*z* of 200 at 30,000 resolution. Data-independent acquisition (DIA) was carried out with sequential isolation windows (width 1.4 *m*/*z*) after each full scan; precursor ions were fragmented in the HCD cell using stepped collision energies of 25, 30, and 35 eV. The automatic gain control (AGC) target was set to 3 × 10^6^, and the maximum injection time was set to Auto for optimal efficiency.

#### 4.3.3. Mouse Protein Database Search and Quantification

DIA raw data were processed in Spectronaut (v16.0, Biognosys AG) using the built-in Pulsar search engine with default settings. The search database was based on the UniProt mouse proteome (Mus_musculus_10090_SP_20220107.fasta; 17,097 sequences). To ensure data quality, the following filters were applied: a 1% false discovery rate (FDR) at both the peptide-spectrum match (PSM) and protein levels, and a requirement of at least one unique peptide per protein. Quantitative analysis was conducted with the MSstats R package (v4.0) using global median normalization for cross-sample intensity comparisons.

### 4.4. Transcriptomic Analysis of Human Public Datasets for Cross-Species Validation

We conducted an analysis of the human anaphylaxis transcriptomic dataset GSE69063, obtained from the GEO database and originally published by Bosco et al. The initial dataset comprised 153 peripheral blood mononuclear cell (PBMC) samples. Following the exclusion of samples related to trauma and sepsis, the dataset was refined to include 33 anaphylaxis patients and 33 healthy control subjects. Furthermore, we examined the human acute myocardial infarction (AMI) transcriptomic dataset GSE66360, published by Muse et al. This dataset included 49 AMI patients and 50 healthy controls, with circulating endothelial cells collected within 24 h of symptom onset. Differentially expressed genes (DEGs) were identified using the limma package (v3.52.4) with thresholds of |log_2_FC| > 0.58 and adjusted *p* < 0.05.

### 4.5. Parallel Reaction Monitoring (PRM) Validation

To validate the differential protein expression profiles, targeted quantitative analysis of mouse serum samples was carried out using parallel reaction monitoring (PRM). Following standardized protein extraction and tryptic digestion (as described above), the resulting peptides were reconstituted in mobile phase A (0.1% formic acid, 2% acetonitrile) and separated on an EASY-nLC 1200 UHPLC system. Separation was achieved with mobile phase B (0.1% formic acid, 90% acetonitrile) using the following gradient: 0–40 min, 6–20% B; 40–52 min, 20–28% B; 52–56 min, 28–80% B; 56–60 min, 80% B, at a flow rate of 500 nL/min. Peptides were ionized via nano-electrospray ionization (nano-ESI, 2.1 kV) and analyzed on a Q Exactive HF-X mass spectrometer. Full-MS scans were acquired at a resolution of 120,000 over an *m*/*z* range of 273–1174, and MS/MS scans were performed at 30,000 resolutions. Data-independent acquisition (DIA) was conducted with higher-energy collisional dissociation (HCD) fragmentation at 28% normalized collision energy. Instrumental parameters were set as follows: full-MS–automatic gain control (AGC) target 3 × 10^6^, maximum injection time (IT) 50 ms; MS/MS–AGC target 2 × 10^5^, maximum IT 220 ms, with 1.4 *m*/*z* isolation windows.

### 4.6. Enzyme-Linked Immunosorbent Assay (ELISA) Validation

Mouse serum samples were analyzed in triplicate using a commercial ELISA kit (Shanghai Ke Shun Biotechnology Co., Ltd., Shanghai, China) according to the manufacturer’s instructions. Briefly, standards, diluted samples, and blank controls were added to the microplate, followed by overnight incubation at 4 °C with HRP-conjugated detection antibody. After washing, substrate solution was added and incubated in the dark at 37 °C for 15 min. The reaction was terminated, and absorbance was measured at 450 nm. Protein concentrations were determined by four-parameter logistic regression based on the standard curve.

### 4.7. Human ASD and SD-CHD Cohorts

This study retrospectively analyzed 20 post-mortem cases obtained from the Forensic Center of Harbin Medical University. Inclusion criteria were: (1) age ≥ 18 years; (2) confirmed diagnosis based on comprehensive autopsy and histopathological examination, classified as either allergic sudden death (ASD group, *n* = 5) or sudden coronary heart disease death (SD-CHD group, *n* = 5); (3) availability of complete cardiac and pulmonary tissue samples. Additional comparison groups comprised ASD with coronary atherosclerosis (ASD + CAS group, *n* = 5) and accidental death cases as controls (Control group, *n* = 5). All cases were blindly verified for demographic characteristics (age and sex; Table 1 and Table 2).

SD-CHD was diagnosed based on the presence of severe coronary artery stenosis (>75%). In cases with survival time >6 h, diagnosis was supported by morphological evidence of acute myocardial ischemia (e.g., myocardial waviness, neutrophilic infiltration, coagulation necrosis). In hyperacute deaths (<6 h), where definitive acute morphological changes may be absent, diagnosis relied on the presence of severe coronary stenosis and the exclusion of other causes of death. Cases with a history of antibiotic exposure but lacking definitive morphological evidence of myocardial ischemia presented a diagnostic challenge between SD-CHD and anaphylactic death, which this study aims to address through molecular biomarker identification.

Tissue samples were fixed in 4% paraformaldehyde for subsequent histopathological analysis. The use of archived paraffin-embedded tissue blocks for this study was approved by the Ethics Committee of Harbin Medical University (No. HMUIRB2022007, 13 June 2022). This approval was based on the clause within the original autopsy consent forms, in which the next of kin consented to the retention and potential future use of tissue samples for scientific research.

### 4.8. Histopathological Validation

Histopathological analysis was performed on mouse and human cardiac and lung (bronchiolar) tissues. After 24 h fixation in 4% paraformaldehyde, tissues were dehydrated in graded ethanol, cleared in xylene, paraffin-embedded, and sectioned at 4 μm. Mouse aortic root plaque burden was quantified as lumen stenosis rate from H&E-stained sections (40×). To validate proteomic findings, IHC was performed using antibodies against FN1, PF4, and GP1BA (1:200, Affinity Biosciences Co., Cincinnati, OH, USA) on mouse and human tissues. Sections were deparaffinized, antigen-retrieved (citrate buffer, pH 6.0), blocked (H_2_O_2_/goat serum), and incubated overnight with primary antibodies at 4 °C, followed by secondary antibody, DAB development, and counterstaining. Plaque burden and protein expression (positive rate) were analyzed using ImageJ 1.54d (National Institutes of Health, Bethesda, MD, USA).at 40× and 400×, respectively.

### 4.9. Statistical Analysis

Bioinformatic analyses were performed using R (v4.3.2). Principal component analysis (PCA) was conducted with the FactoMineR (v2.6) and factoextra (v1.0.7) packages. Differential expression patterns were visualized using ggplot2 (v3.4.2) for volcano plots and pheatmap (v1.0.12) for hierarchical clustering. Functional enrichment analysis for Gene Ontology (GO) terms and KEGG pathways was performed using clusterProfiler (v4.0.5).

For conventional statistical testing, all data were processed and visualized with GraphPad Prism v10.1.2 (GraphPad Software, Boston, MA, USA). Normality of continuous variables was assessed using the Shapiro–Wilk test, and homogeneity of variance was evaluated with Levene’s test. When both assumptions were satisfied, data were expressed as mean ± standard deviation (mean ± SEM). Comparisons between two groups were performed using independent-samples *t*-tests, and comparisons among multiple groups were conducted with two-way analysis of variance (two-way ANOVA). If a significant interaction effect was detected, Tukey’s post -hoc test was used for multiple comparisons. Categorical data were summarized as frequencies (percentages). Between-group comparisons were carried out with the chi-square (*χ*^2^) test or Fisher’s exact test when expected frequencies were <5. A two-tailed significance level of *α* = 0.05 was adopted, and *p* < 0.05 was considered statistically significant.

## 5. Conclusions

Through an innovative integrative strategy, this study systematically reveals the differential expression profiles of FN1, PF4, and GP1BA, which show high discriminatory value between ASD and SD-CHD. The multimarker, multilevel (mouse serum and tissue) diagnostic framework proposed here provides a novel molecular-pathology solution that combines scientific rigor with practical applicability to address this long-standing diagnostic challenge in forensic practice.

## Figures and Tables

**Table 1:** Demographic characteristics of human postmortem samples.

Case No.	Gender	Age (Years)	Cause of Death
**Control Group**			
1	Male	45	Severe craniocerebral injury
2	Male	45	Intestinal rupture
3	Male	52	Fall from height
4	Female	53	Hepatic rupture
5	Female	26	Severe craniocerebral injury
**SD-CHD Group**			
6	Male	49	Coronary heart disease
7	Male	38	Coronary heart disease
8	Male	58	Coronary heart disease
9	Female	51	Coronary heart disease
10	Female	55	Coronary heart disease
**ASD Group**			
11	Male	39	Anaphylactic
12	Male	57	Anaphylactic
13	Female	47	Anaphylactic
14	Female	50	Anaphylactic
15	Female	35	Anaphylactic
**ASD + CAS Group**			
16	Male	37	Anaphylactic (with coronary atherosclerosis)
17	Male	58	Anaphylactic (with coronary atherosclerosis)
18	Male	42	Anaphylactic (with coronary atherosclerosis)
19	Female	53	Anaphylactic (with coronary atherosclerosis)
20	Female	42	Anaphylactic (with coronary atherosclerosis)

**Table 2:** The age and sex demographic characteristics of the human postmortem samples.

	Control	SD-CHD	ASD	ASD + CAS	*p* Value
Age (years)	44.20 ± 10.85	50.20 ± 7.662	45.60 ± 8.764	46.40 ± 8.735	>0.05
**Gender**					
Male	3	3	2	3	>0.05
Female	2	2	3	2	

## Data Availability

The data supporting the study findings is available upon request from the corresponding authors.

## Supplementary Materials

The following supporting information can be downloaded at: https://www.mdpi.com/article/10.3390/ijms27052166/s1.

## Abbreviations

The following abbreviations are used in this manuscript:

ASD	Anaphylactic Sudden Death
SD-CHD	Sudden death from coronary heart disease
LC-MS/MS	Liquid Chromatography–Tandem Mass Spectrometry
PRM	Parallel Reaction Monitoring
IHC	Immunohistochemistry
OVA	Ovalbumin
ASA	Active Systemic Anaphylaxis
FN1	Fibronectin 1
GP1BA	Platelet Glycoprotein Ibα Chain
PF4	Platelet Factor 4
CAS	Coronary Atherosclerosis
cTn	Cardiac Troponin
CK-MB	Creatine Kinase-MB
IACUC	Institutional Animal Care and Use Committee
HFD	High-Fat Diet
DTT	Dithiothreitol
IAA	Iodoacetamide
FDR	False Discovery Rate
PSM	Peptide-Spectrum Match
GEO	Gene Expression Omnibus
AMI	Acute Myocardial Infarction
PCA	Principal Component Analysis
GO	Gene Ontology
KEGG	Kyoto Encyclopedia of Genes and Genomes
PPI	Protein–Protein Interaction
NSI	Nano-Electrospray Ionization
HCD	Higher-Energy Collisional Dissociation
AGC	Automatic Gain Control
IT	Injection Time
EDA-FN	EDA-Containing FN1
FC	Fold Change
WT	Wild-Type
AP	Anaphylaxis
AS	Atherosclerosis
