# Supplementary material for: Integrative Proteomic and Transcriptomic Profiling Identifies Candidate Biomarkers for Discriminating Anaphylactic from Cardiac Sudden Death
Source: Int J Mol Sci. 2026 Feb 25;27(5):2166. doi: 10.3390/ijms27052166 (PMC12984341; doi:10.3390/ijms27052166)
Supplement: Supplementary file 1 [file ijms-27-02166-s001.zip › ijms-4131541-supplementary/Figure S2.pdf]

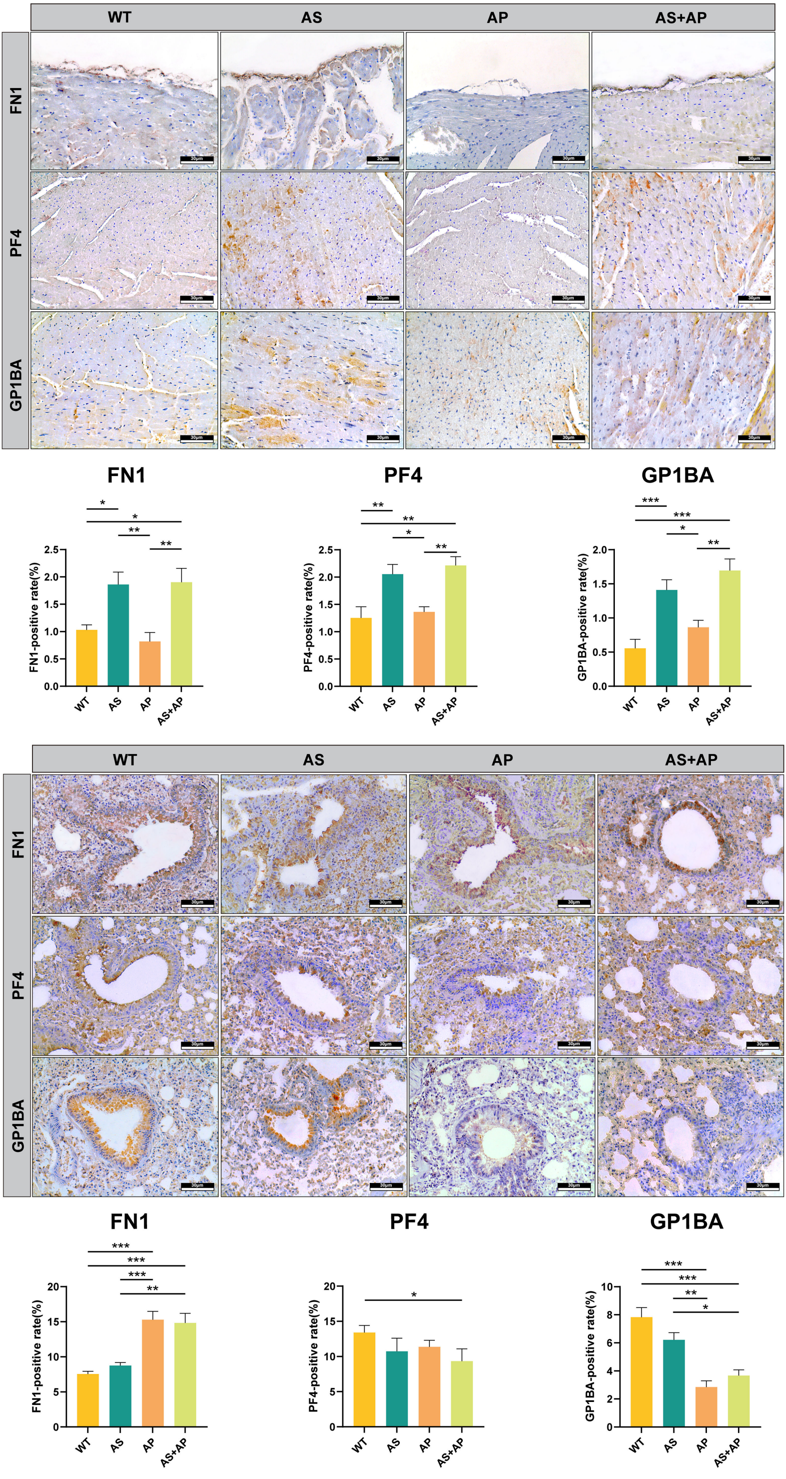

FigureS2 Immunohistochemical validation of marker protein expression in murine tissues. Rep-resentative IHC staining of FN1, PF4, and GP1BA in myocardial tissue (A) and airway epithelium(B) from WT, AS, AP, and AS+AP groups (bar: 30 μm). Quantification data are presented as mean ± SEM (A, B). *$p < 0.05$, **$p < 0.01$, ***$p < 0.001$.
