# Supplementary material for: Integrative Proteomic and Transcriptomic Profiling Identifies Candidate Biomarkers for Discriminating Anaphylactic from Cardiac Sudden Death
Source: Int J Mol Sci. 2026 Feb 25;27(5):2166. doi: 10.3390/ijms27052166 (PMC12984341; doi:10.3390/ijms27052166)
Supplement: Supplementary file 1 [file ijms-27-02166-s001.zip › ijms-4131541-supplementary/Table S1.pdf]

Table S1. MS/MS spectrum database search analysis summary

Category	Number	FDR cut-off
Identified peptides	9055	<0.01
Identified proteins	1325	<0.01
Comparable proteins	1317	<0.01

Proteins were identified with at least one unique peptide.
