# Supplementary figures and images for: Integrative Proteomic and Transcriptomic Profiling Identifies Candidate Biomarkers for Discriminating Anaphylactic from Cardiac Sudden Death
Source: Int J Mol Sci. 2026 Feb 25;27(5):2166. doi: 10.3390/ijms27052166 (PMC12984341; doi:10.3390/ijms27052166)

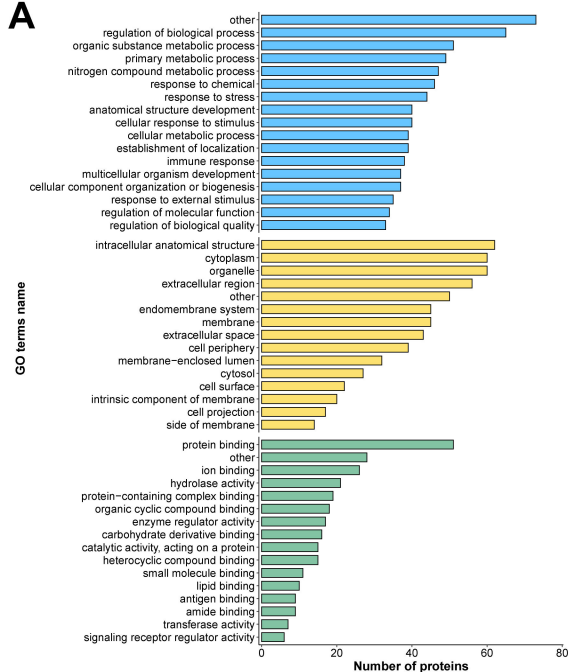

**B**

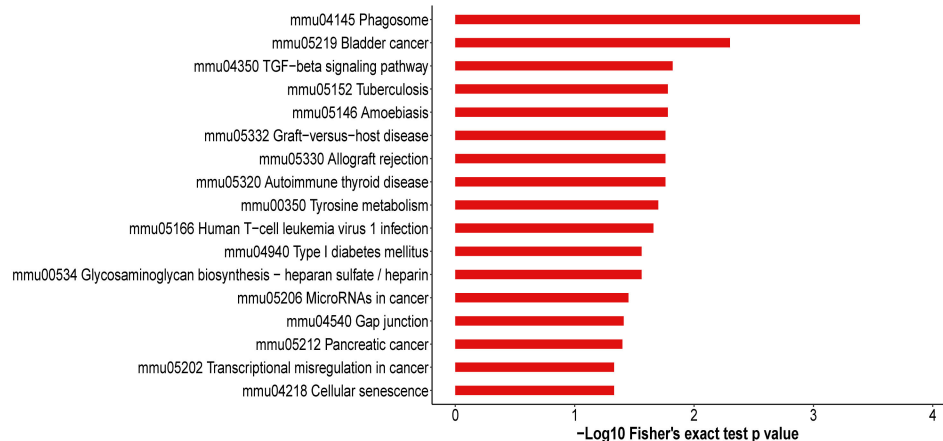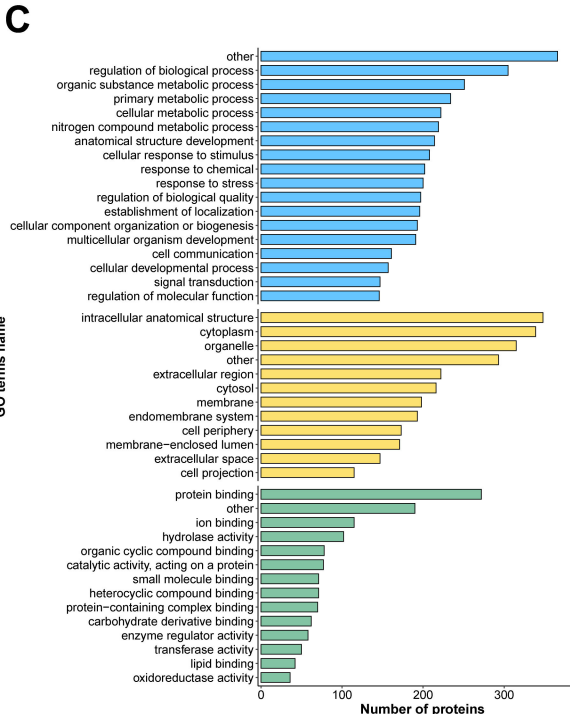

**D**

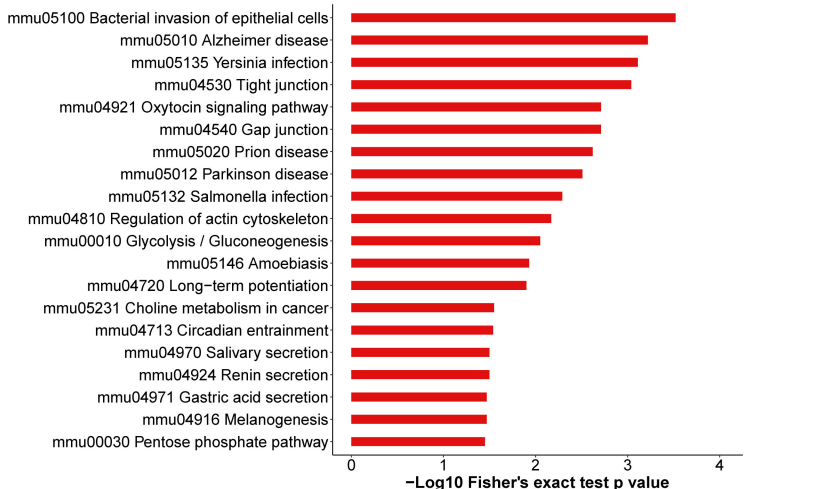

Supplement: Supplementary file 1 [file ijms-27-02166-s001.zip › ijms-4131541-supplementary/Figure S1.pdf]
